# An Unexpected Activity of a Minor Cannabinoid: Cannabicyclol (CBL) Is a Potent Positive Allosteric Modulator of Serotonin 5-HT_1A_ Receptor

**DOI:** 10.1021/acs.jnatprod.4c00977

**Published:** 2025-01-15

**Authors:** Mehdi Haghdoost, Yvonne DePorre, Max Figi, Scott Young, Caitlyn Krebs, Marcel O. Bonn-Miller

**Affiliations:** † Nalu Bio Inc., 38 Keyes Avenue, Suite 117, San Francisco, California 94129, United States; ‡ Charlotte’s Web, 700 Tech Court, Louisville, Colorado 80027, United States

## Abstract

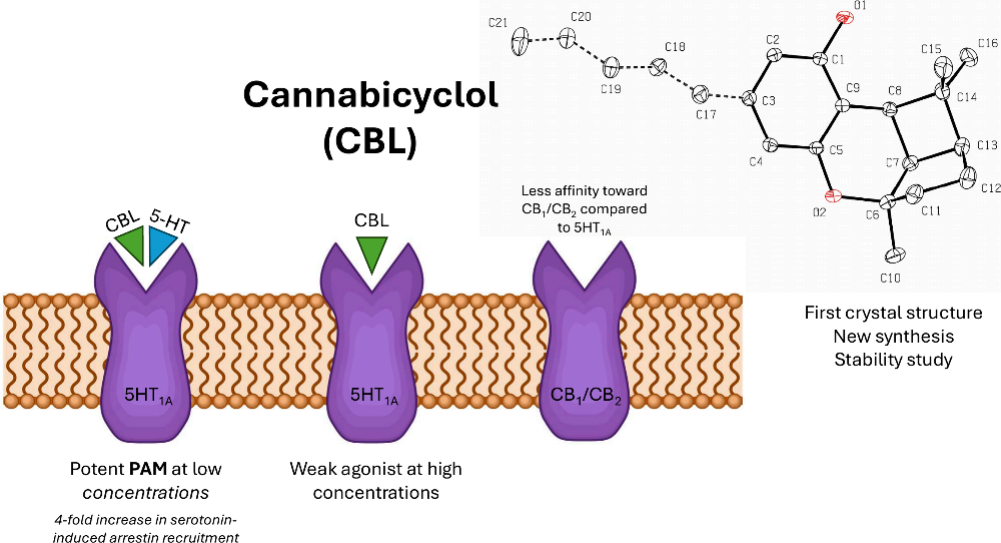

Cannabicyclol ((±)-CBL), a minor phytocannabinoid, is largely unexplored, with its biological activity previously undocumented. We studied its conversion from cannabichromene (CBC) using various acidic catalysts. Montmorillonite (K30) in chloroform at room temperature had the highest yield (60%) with minimal byproducts. Key reaction conditions, such as solvent, temperature, and time, significantly impacted the yield. The structure of (±)-CBL was confirmed via X-ray crystallography. Stability studies showed that (±)-CBL and its MCT oil dilution remain stable at 25–40 °C for three months. Radioligand binding assays revealed high affinity of CBL for the 5-HT_1A_ receptor but weak interaction with CB_1_ and CB_2_ receptors. At 10 μM and 1 μM, (±)-CBL inhibited [^3^H]-8-hydroxy-DPAT binding to 5-HT_1A_ by 75% and 20%, respectively. Functional assays showed that (±)-CBL acts as a weak agonist at high concentrations but a potent positive allosteric modulator of serotonin-induced activation at low concentrations. At 4 μM, (±)-CBL increased serotonin-induced β-arrestin recruitment from 20% to 80%. This unique modulatory profile highlights the potential of (±)-CBL in drug discovery targeting serotonin receptors.

The cannabis plant produces a complex mixture of phytochemicals, including phytocannabinoids and terpenes.^1^ The overall effect of cannabis is defined not only by the individual impact of each of these chemicals but also by how they modulate one another’s effects.^2^ The human impact of the two main constituents of cannabis, tetrahydrocannabinol (THC)^3^ and cannabidiol (CBD),^4^ has been extensively discussed and reviewed in the literature. Recent studies have also started to explore the effects of minor cannabinoids such as tetrahydrocannabivarin (THCV),^5,6^ cannabinol (CBN),^7,8^ cannabigerol (CBG),^9−11^ and cannabichromene (CBC).^12^ However, cannabis can contain a plethora of other compounds, many of which have largely unknown biological activities. This lack of comprehensive data on the full spectrum of cannabis constituents poses a significant hurdle in fully understanding the therapeutic potential and safety profile of cannabis products. Further research is essential to elucidate the roles and effects of these minor cannabinoids and other phytochemicals, potentially uncovering new therapeutic uses or safety concerns associated with cannabis consumption. However, due to the limited natural quantities of these minor cannabinoids, the discovery of robust chemical synthesis methods is crucial for producing them on a large enough scale to conduct comprehensive biological studies. Chemical synthesis can yield large-scale minor cannabinoids of high purity, allowing consistency and reliability in biological research.

Cannabicyclol (CBL) is one of these understudied phytocannabinoids naturally occurring in cannabis plants. Remarkably, significant quantities of this compound were detected in a 2700-year-old cannabis sample unearthed in China.^13^ However, CBL is not directly synthesized by plant enzymes; instead, it forms through nonenzymatic transformations of other phytocannabinoids, primarily from the thermal and/or photodegradation of cannabichromene (CBC).^14,15^ Despite being a degradation product, the structure of CBL, characterized by three fused rings, is notably distinct from other cannabinoids, leading to the classification of CBL and its derivatives into a unique class of phytocannabinoids known as CBL-type.^16^ The unexpected structure of CBL led to its initial mischaracterization as a THC-like compound,^17^ necessitating multiple revisions to accurately determine its correct structure.

In the study ’Chemical Basis of Hashish Activity,’ Mechoulam reported that CBL at a dose of 5.5 μg/kg administered intravenously does not produce an intoxicating effect in rhesus monkeys. Furthermore, when coadministered with Δ^9^-THC at 250 μg/kg intravenously, CBL at 5.5 μg/kg was not shown to alter the response to Δ^9^-THC.^18^ This study has often been cited to support the anecdotal conclusion that (±)-CBL has no intoxicating effect. While this conclusion may be accurate, it is not a logical deduction from the study, given the very low doses of CBL used. In a 1980 review, Turner^19^ summarized a report from Razdan (1970) that demonstrated piloerection, increased respiration, and touch-induced irritability in mice at a dose of 10 mg/kg of CBL.^20^ From 1970, for more than 50 years, no further studies assessed the biological effect of CBL until a 2024 study reported that CBL inhibits SARS-CoV-2 spike protein-mediated membrane fusion with an EC_50_ of 10.8 μM,^21^ a concentration that is unlikely to be biologically significant. In addition, a recent preprint study proposed CBL as a potential therapeutic candidate for Parkinson’s disease based on findings from in silico analyses.^22^ However, this hypothesis requires validation through further *in vitro* and *in vivo* studies. Despite these limited studies, the biological properties of CBL remain largely unknown. No substantial *in vitro*, *in vivo*, or human studies have provided concrete information about the potential effects of this compound. This knowledge gap underscores the need for comprehensive research to explore the pharmacological and therapeutic potential of CBL, which could reveal new insights into the broader spectrum of cannabis phytochemicals and their impacts on human health.

This research aims to fill the significant gaps in the current understanding of CBL, providing foundational insights into its potential biological activities and therapeutic applications. In this study, we not only improved the synthesis of (±)-CBL and reported its crystal structure and stability for the first time, but we also investigated its affinity and functionality at cannabinoid and serotonin receptors. Among the serotonin receptors, the 5-HT_1A_ receptor has been a particular focus due to its significant role in the biological effects of several cannabinoids, notably CBD. The 5-HT_1A_ receptor, a prominent target in the central nervous system, is implicated in modulating mood, anxiety, and neuroprotection.^23^ CBD has been shown to exert some of its therapeutic effects through partial agonism at the 5-HT_1A_ receptor,^24^ contributing to its anxiolytic,^25^ antidepressant,^26^ and anticonvulsant^27^ properties. This receptor’s ability to influence such biological responses positions it as an important target in cannabinoid research, where rare cannabinoids like CBL may exhibit therapeutic potential through similar mechanisms.

## Results and Discussion

### Synthesis

Two primary reactions have been studied in the conversion of (±)-CBC to (±)-CBL. The first involves using a homogeneous acid, such as trifluoroacetic acid (TFA), for acid-catalyzed ring-closing.^28^ Additionally, iron-catalyzed ring-closing has recently emerged as an effective method for synthesizing CBL-based molecules.^29^ The first synthesis of CBL was conducted by exposing CBC to UV light, which led to the untested hypothesis that the presence of CBL in the cannabis plant results from the natural exposure of CBC to light.^30^ In this study, we investigated the use of heterogeneous acids as novel catalysts for the synthesis of the CBL molecule (Table 1).

**Table 1:**
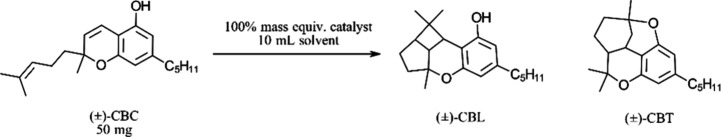
Conversion of (±)-CBC to (±)-CBL and (±)-CBT

Entry	Catalyst	Solvent	Temp. (°C)	Time (h)	CBC conversion (%)	(±)-CBL yield (%)	CBT yield (%)
1	ZSM-5 (MR38)	heptane	0	24	28	1	2
2	ZSM-5 (MR38)	heptane	65	24	37	1	2
3	SSZ-13	heptane	0	24	27	1	3
4	Al-MCM-41	heptane	0	24	21	1	3
5	Amberlyst 15	heptane	0	2	46	0	8
6	Montmorillonite (K30)	heptane	0	24	40	8	1
7	Montmorillonite (K30)	chloroform	0	2	27	25	2
8	Montmorillonite (K30)	chloroform	0	24	41	32	2
9	Montmorillonite (K30)	chloroform	22	24	80	60	2
10	Montmorillonite (K30)	TBME	0	24	22	1	0
11	Montmorillonite (K30)	acetic acid	0	24	46	4	0
12	pTsOH	chloroform	0	2	45	0	9
13	CSA	chloroform	0	2	86	0	21

The fact that the conversion of (±)-CBC to (±)-CBL is promoted by homogeneous acids prompted us to explore heterogeneous catalysts with varying Lewis/Bronsted acidity and structural characteristics. In our selection process, we also considered factors such as commercial availability, cost, and the potential for industrial application of each catalyst. Accordingly, various acidic heterogeneous catalysts, including amberlyst 15, ZSM-5 (MR38), SSZ-13, AlMCM-41, and montmorillonite, were screened for their efficiency in catalyzing this reaction. Surprisingly, only montmorillonite exhibited a low yield in the formation of (±)-CBL (Table 1, entry 6). Other catalysts, such as ZSM-5, SSZ-13, and Al-MCM-41, facilitated some conversion of (±)-CBC but resulted in a complex mixture of products (Table 1, entries 1–5). This product mixture was not fully characterized; however, in all cases, CBT was identified as one of the products. In some cases, HPLC-DAD analysis revealed small peaks with retention times similar to that of Δ^9^-THC. This peak may be attributed to *iso*-THC, a phytocannabinoid structurally similar to CBT.^31^ Further experiments using homogeneous acids, such as para-toluenesulfonic acid (pTsOH) and camphorsulfonic acid (CSA), also failed to produce (±)-CBL (Table 1, entries 12 and 13). Instead, these acids resulted in complex mixtures containing CBT as a significant component. The distinct catalytic performance of montmorillonite, despite its significantly weaker acidity compared to amberlyst, suggests that factors other than acidity play a crucial role in the conversion of (±)-CBC to (±)-CBL. The presence of iron ions,^32^ known to promote the conversion of (±)-CBC to (±)-CBL,^29^ in the multilayer structure of montmorillonite might be the key catalytic factor. Further studies using iron-doped catalysts could provide valuable insights and support for this hypothesis.

The conversion of (±)-CBC to (±)-CBL using 100% mass equivalent of montmorillonite (K30) in chloroform yielded a 27% conversion rate for (±)-CBC (Table 1, entry 7). This process resulted in a 25% yield of (±)-CBL and a 2% yield of (±)-CBT after 2 h at 0 °C. No significant formation of other byproducts was detected during this reaction period. When the reaction time was extended to 24 h, the yield of (±)-CBL increased to 32% (Table 1, entry 8). Further optimization by increasing the reaction temperature to room temperature (∼22 °C) significantly improved the yield, achieving 60% (±)-CBL within 24 h (Table 1, entry 9). Among the solvents tested, chloroform proved to be the most effective. Reactions conducted in *tert*-butyl methyl ether (TBME) and acetic acid did not show significant (±)-CBL formation (Table 1, entries 10 and 11). In heptane, only minimal activity was observed at 0 °C (Table 1, entry 6). These findings underscore the importance of optimizing reaction conditions, such as solvent choice, temperature, and reaction time, to maximize the yield of (±)-CBL.

Our synthesis method for CBL offers distinct advantages over previously reported methods, such as the homogeneous acid catalysis by Yeom et al.^28^ and the iron-mediated reaction by Li et al.^29^ Notably, our approach utilizes montmorillonite, a heterogeneous catalyst that can be easily separated from the reaction mixture through simple filtration, enhancing practicality and reducing waste associated with catalyst recovery. Unlike prior protocols, which employed liquid–liquid extraction workups and chromatography for CBL purification, our method allows for a straightforward crystallization of CBL directly from the reaction mixture after catalyst removal. This streamlined process simplifies purification, potentially increasing efficiency. However, it is important to note that our synthesis, owing to the heterogeneous reaction setup, requires a longer reaction time (24 h) compared to the faster TFA-catalyzed (1.5 h) and iron-mediated synthesis (12 h). Notably, despite this extended reaction duration, the CBL yield remains comparable to those achieved by previously reported methods.

It is important to note that the CBC used in this study was racemic (±)-CBC due to the challenging isolation of enantiopure CBC and the potential for spontaneous racemization of CBC to a scalemic mixture.^33^ The stereochemistry of the newly formed five- and four-membered rings in the CBL structure is determined by the facial arrangement of the isoprenyl unit of CBC, leading exclusively to the formation of *cis*-fused rings. As a result, the CBL produced from (±)-CBC maintains the chirality of the starting material, yielding a racemic mixture of (±)-CBL enantiomers without generating diastereomers. This was confirmed through NMR analysis, which indicated the presence of a single diastereomer. Although recent studies have demonstrated the separation of (+)- and (−)-CBL using polysaccharide chiral stationary phases,^34^ due to the complexity of conducting the chiral purification at a large scale, we opted to use a racemic mixture of (±)-CBL for our stability and *in vitro* studies.

### Crystal Structure

**Figure 1:**
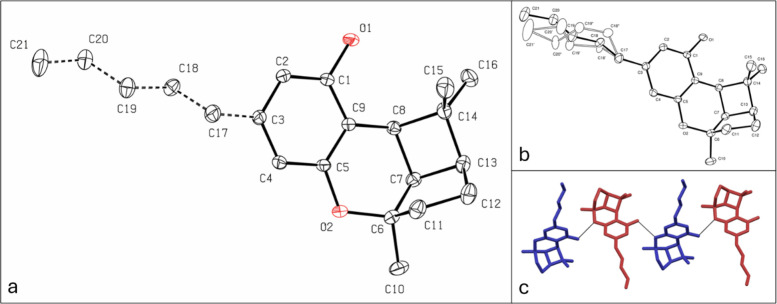
(a) ORTEP drawings of (±)-CBL X-ray structure. Ellipsoids are at the 50% probability level, and hydrogen atoms are omitted for clarity. (b) ORTEP drawings of the same molecule with disordered minor conformers depicted with hollow ellipsoids and bonds. (c) Orientation of (±)-CBL molecules in the crystal packing.

Another intriguing aspect of the reaction in chloroform is that (±)-CBL can be easily crystallized from the reaction mixture by cooling. Slow cooling of the mixture results in high-quality crystals suitable for X-ray crystallographic analysis. X-ray analysis confirmed the structure of (±)-CBL, revealing an uncommon tricyclic arrangement consisting of fused 4-, 5-, and 6-member rings (Figure 1a). The *n*-pentyl tail displayed disorder, with the chain adopting three distinct conformations. This suggests the presence of multiple energetically favorable conformations within the crystal lattice. Figure 1a presents the normalized occupancies of the *n*-pentyl carbon atoms, while Figure 1b illustrates the different conformations. In (±)-CBL monoclinic P 2_1_/n crystal structure, (±)-CBL molecules are packed in antiparallel orientation thanks to the hydrogen bonds between the phenolic OH group and the oxygen of the tetrahydropyran (Figure 1c). The crystal structure of a few CBL derivatives, such as dibromocannabicyclol,^35^ has been previously reported. However, to the best of our knowledge, this study presents the first reported structure of CBL.

### Stability

**Figure 2:**
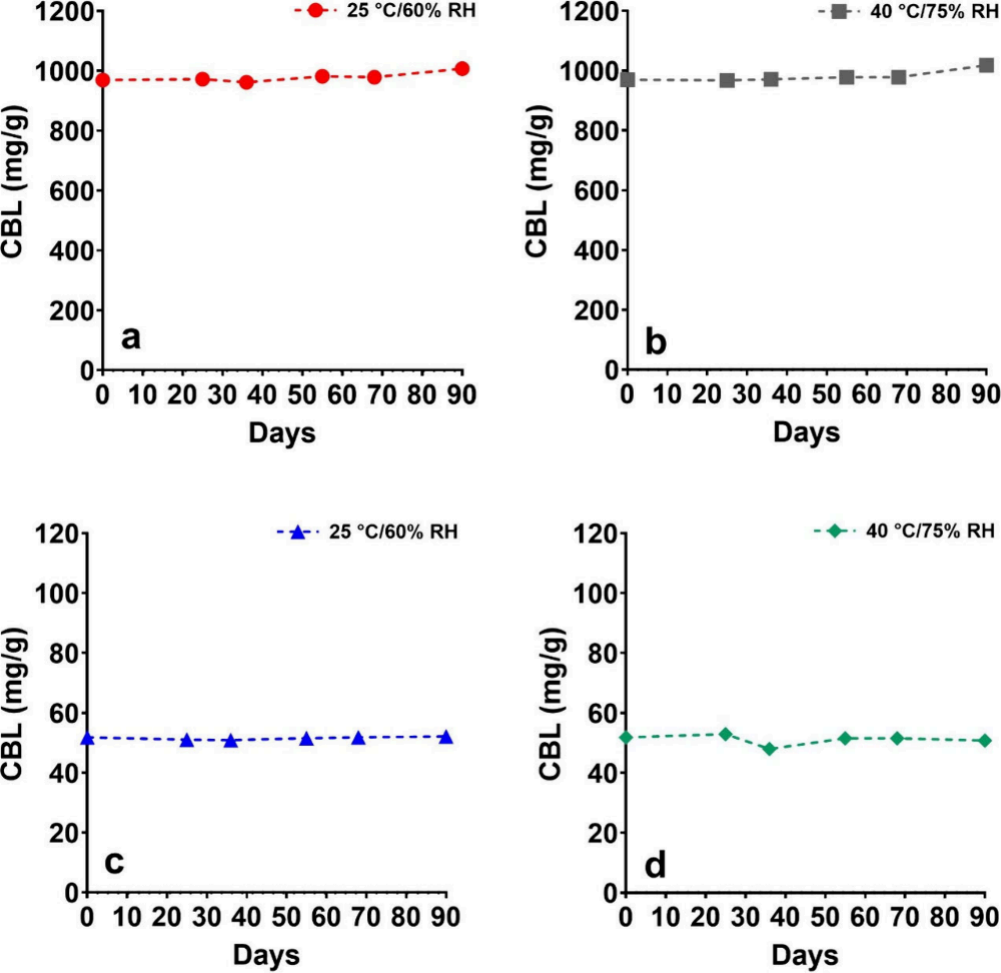
Stability of (a, b) (±)-CBL isolate and (c, d) 50 mg/g tincture in MCT oil at (a, c) 25 °C/60% RH and (b, d) 40 °C/75% RH. Each time point was measured from a single sample in three analytical replicates.

Most cannabinoids suffer from long-term stability challenges, such as susceptibility to oxidation, thermal degradation, and isomerization under acidic conditions.^36^ CBD is prone to these degradation pathways.^37−39^ To address this issue, a preliminary stability study was conducted on (±)-CBL to ensure its stability under storage and test conditions before any *in vitro* experiments. The stability study evaluated both (±)-CBL isolate (>98% purity) and a 50 mg/g (±)-CBL tincture in medium-chain triglyceride (MCT) oil, a commonly used formulation oil for cannabinoids.^40^ The study was performed over two months under standard conditions (25 °C and 60% relative humidity) and accelerated conditions (40 °C and 75% relative humidity). The results demonstrated no signs of degradation under any tested conditions, indicating that (±)-CBL, both as an isolate and in MCT oil solution, is stable within the tested time frame (Figure 2). Additionally, unlike most cannabinoids, which exhibit color changes due to the formation of cannabiquinones during storage,^41^ (±)-CBL showed no such color change. The absence of degradation within the study’s time frame precluded the calculation of a precise shelf life for (±)-CBL. However, the findings suggest that (±)-CBL possesses a very good stability profile, although longer-term studies following ICH guidelines for stability are needed to accurately determine its shelf life.

It is important to note that the (±)-CBL isolate used in the stability study contained approximately 1% w/w THC (10 mg/g), likely in the form of *iso*-THC, as the main impurity. Notably, the quantity of this impurity remained unchanged throughout the study period. This small quantity of THC was not expected to affect the *in vitro* analysis. However, to ensure that the *in vitro* data is solely driven by CBL, we utilized highly purified (±)-CBL Certified Reference Material (CRM) devoid of any detectable cannabinoid impurities for the following in vitro experiments. The use of CBL CRM, with its verified purity, allowed us to confidently attribute the observed biological effects exclusively to CBL, eliminating any potential confounding influence from THC or other cannabinoids.

### Radioligand Binding Assay

**Figure 3:**
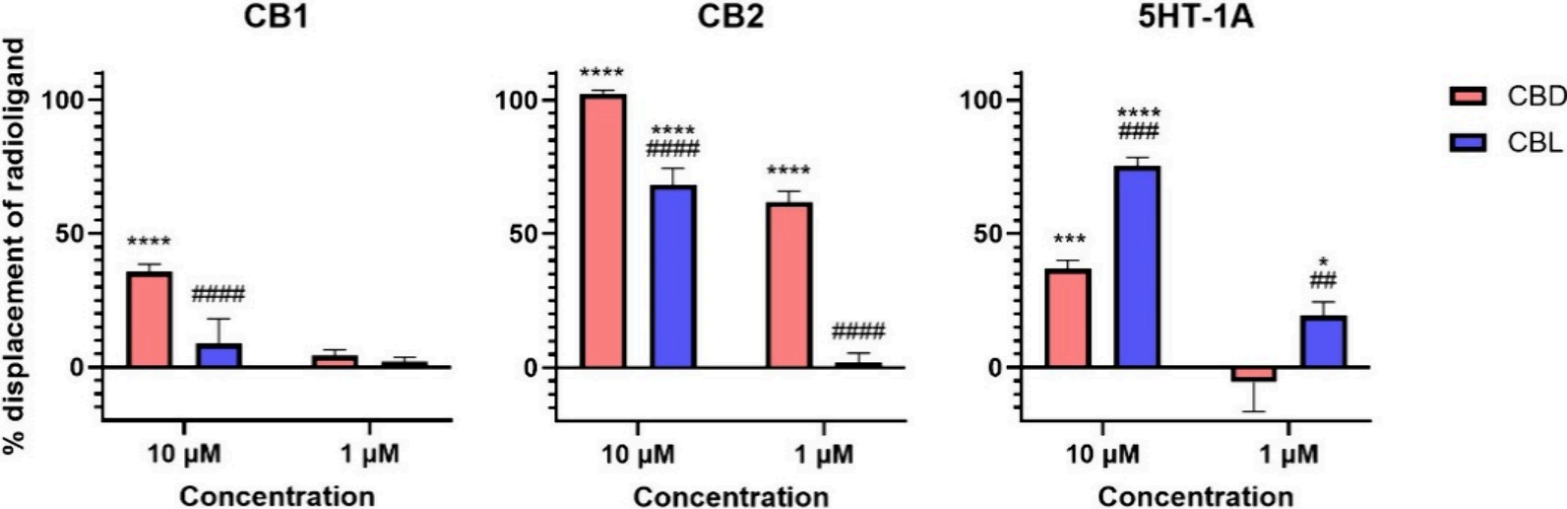
Displacement of radiolabeled [^3^H]-CP55940 (CB_1_ and CB_2_) and [^3^H]-8-hydroxy-DPAT (5-HT_1A_) by cold (±)-CBL and CBD at 1 and 10 μM. One hundred percent relative activity was normalized to maximum replacement by high control (5 μM cold CP55940 for CB_1_, 1 μM WIN55212-2 for CB_2_, and 0.3 μM serotonin for 5-HT_1A_); 0% relative activity to compound vehicle control. ****p < 0.0001, ***p < 0.001, *p < 0.05 versus vehicle control. ^####^*p* < 0.0001, ^###^*p* < 0.001, ^##^*p* < 0.01 versus CBD. Error bars represent the standard deviation of three independent measurements. CBD data have been taken from our previous publication.^43^

We investigated the interaction of (±)-CBL with the cannabinoid receptors CB_1_ and CB_2_, as well as the serotonin 5-HT_1A_ receptor, utilizing radioligand binding assays (Figure 3). The cannabinoid receptors CB_1_ and CB_2_ are well-documented primary targets of cannabinoids,^42^ while the 5-HT_1A_ receptor is considered a significant biological target of CBD.^24^ At both 10 μM and 1 μM concentrations, (±)-CBL did not inhibit the binding of radiolabeled CP55940 to the CB_1_ receptor, indicating a lack of affinity toward this receptor. Conversely, (±)-CBL demonstrated a 68% inhibition of CP55940 binding to the CB_2_ receptor at 10 μM, although no inhibition was detected at the 1 μM concentration. Notably, (±)-CBL exhibited substantial inhibitory activity on the 5-HT_1A_ receptor, inhibiting the binding of radiolabeled 8-OH-DPAT by an average of 75% at 10 μM and 20% at 1 μM. Comparative analysis with CBD under similar conditions^43^ indicated that (±)-CBL has a lower affinity for cannabinoid receptors but a markedly higher affinity for the 5-HT_1A_ receptor than CBD. Based on the inhibitory effect observed at 1 μM for (±)-CBL, the 5-HT_1A_ receptor can be considered one of the main potential biological targets of (±)-CBL. This unexpected activity of (±)-CBL at the 5-HT_1A_ receptor, unique among tested phytocannabinoids, warrants further investigation.

### *In Vitro* Functionality Assay

**Figure 4:**
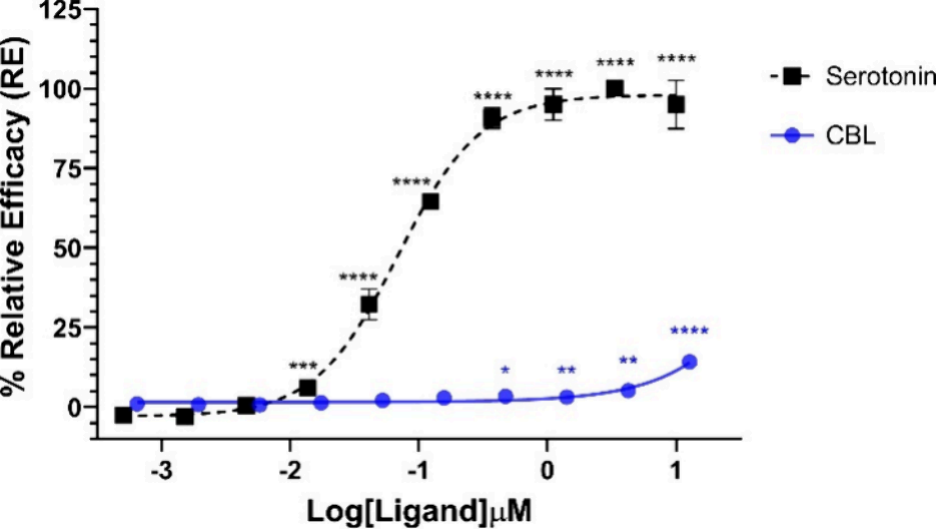
Agonist 5-HT_1A_ β-arrestin assay for endogenous ligand (serotonin) and (±)-CBL after 90 min of incubation time using the PathHunter arrestin assay. One hundred percent relative activity was normalized to maximum stimulation of serotonin and 0% relative activity to compound vehicle control. Error bars represent the standard deviation of three independent measurements. ****p < 0.0001, ***p < 0.001, **p < 0.05, *p < 0.01 versus vehicle control.

We initially investigated the agonist activity of (±)-CBL at the 5-HT_1A_ receptor using a β-arrestin PathHunter assay (Figure 4). For this purpose, CHO-K1 cells overexpressing PK-tagged human 5-HT_1A_ receptors were treated with a range of (±)-CBL concentrations (12 μM - 0.64 nM), and β-arrestin recruitment was measured using a chemiluminescence assay. Our findings indicated that (±)-CBL demonstrated minimal agonist activity at concentrations above 0.5 μM, achieving only 15% of the efficacy of serotonin at the highest tested concentration of 12 μM. When compared to the previously reported activity of cannabidiol (CBD) in the same assay,^43^ the agonist activity of (±)-CBL was notably weaker, although radioligand binding assays revealed that (±)-CBL exhibited higher affinity toward the 5-HT_1A_ receptor than CBD. The weak agonist activity of (±)-CBL was further evidenced by its failure to reach an EC_50_ value, in contrast to serotonin, which produced an EC_50_ of 69 nM in this assay.

**Figure 5:**
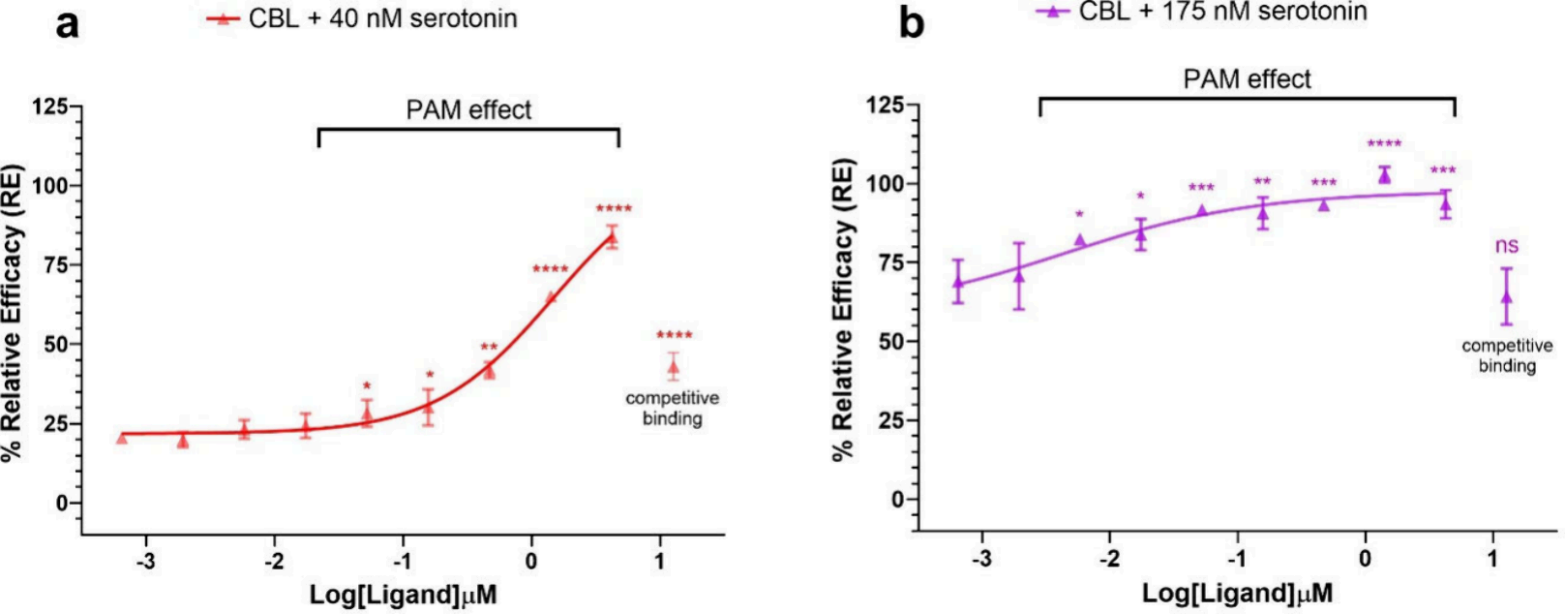
Normalized response in the 5-HT_1A_ PathHunter arrestin assay for (±)-CBL after (a) 40 nM or (b) 175 nM addition of serotonin (endogenous agonist). One hundred percent relative activity was normalized to maximum stimulation of serotonin and 0% relative activity to vehicle control. ****p < 0.0001, ***p < 0.001, **p < 0.05, *p < 0.01 versus vehicle control. Error bars represent the standard deviation of three independent measurements.

To further investigate the interaction of (±)-CBL with the 5-HT_1A_ receptor, we examined how (±)-CBL could potentially modulate the response of serotonin at this receptor. For this purpose, we first treated a 5-HT_1A_-expressing CHO-K1 cell line with 0.6 nM to 12 μM concentrations of (±)-CBL for 30 min, followed by the addition of either 175 nM or 40 nM of serotonin and monitored the β-arrestin recruitment using a chemiluminescence assay. The serotonin concentrations of 175 nM and 40 nM were selected based on serotonin EC_80_ and EC_20_ values, respectively. We observed unexpected results: when coincubated with 40 nM of serotonin, (±)-CBL at a concentration as low as 50 nM significantly enhanced receptor activation with serotonin (Figure 5a). Increasing the dose of (±)-CBL further increased serotonin-induced receptor activation in a dose-dependent manner, reaching over 80% receptor activation when serotonin was incubated with 4 μM of (±)-CBL. Considering that (±)-CBL by itself is a weak agonist of the receptor, this data clearly indicates that (±)-CBL acts as a positive allosteric modulator (PAM) for serotonin. Interestingly, when the (±)-CBL concentration was further increased to 12 μM, the PAM effect was diminished. This aligns with the radioligand binding assay (Figure 4), which shows significant competitive binding of (±)-CBL at a 10 μM concentration, suggesting that at very high concentrations, (±)-CBL may hinder serotonin from effectively binding to the active site of the receptor.

The PAM effect of (±)-CBL was even more pronounced at higher serotonin concentrations. At a low concentration of 5 nM, (±)-CBL significantly enhanced the β-arrestin recruitment of 175 nM serotonin. Co-incubation of 175 nM serotonin and 1 μM (±)-CBL resulted in full activation of the receptor, an effect that neither 175 nM serotonin nor 1 μM (±)-CBL could achieve alone. However, similar to our previous observations, increasing the (±)-CBL concentration above 10 μM diminished the PAM effect. This reduction is due to the competitive binding of (±)-CBL at high concentrations, which can interfere with the ability of serotonin to effectively bind to the receptor.

It has been established that some endocannabinoids and synthetic cannabinoids can act as allosteric modulators of serotonin 5HT receptors.^44^ For example, studies have highlighted the role of oleamide, an endogenous ligand of cannabinoid receptors, and several other long-chained fatty acid amides as allosteric modulators of 5-HT receptors.^45^ Synthetic cannabinoids such as AM2201 and JWH-018 have also been reported to exhibit positive allosteric modulation effects at the 5-HT_1A_ receptor. Specifically, these two compounds enhance the maximal effect of serotonin in activating the Gi pathway by 21% and 12%, respectively, at a concentration of 10 μM.^46^ Interestingly, positive allosteric modulation of 5-HT_1A_ has not been reported for phytocannabinoids extracted from *Cannabis sativa L.* For instance, Δ^9^-THC failed to show any PAM activity under conditions similar to those tested for AM2201 and JWH-018.^46^ This highlights the unique profile of (±)-CBL, a cannabinoid with a distinct allosteric modulatory effect on the 5-HT_1A_ receptor, setting it apart from other phytocannabinoids.

Using dose–response curve fitting, we calculated EC_50_ values of 5 nM and 1480 nM for the allosteric effect of (±)-CBL at 40 nM and 175 nM concentrations of serotonin, respectively. These data enable us to characterize (±)-CBL as one of the most potent PAMs of the 5-HT_1A_ receptor. Cannabinoids, due to their favorable safety profile, can be administered at high doses without significant adverse effects. For instance, the *C*_max_ of CBD in human blood/plasma samples after a single high-dose administration can exceed 3 mM.^47^ This property suggests that the doses and activity observed in this study for (±)-CBL are biologically relevant. Serotonin receptor allosteric modulation is an innovative targeting approach that confers specific advantages in distinguishing between highly homologous receptor subtypes and is considered a viable drug discovery strategy.^48−53^ Among serotonin receptors, 5-HT_1A_, which is regarded as the most widespread of all the 5-HT receptors,^54^ plays a crucial role in neuromodulation and is a target of many mental health medications. However, 5-HT_1A_ allosteric modulators have not been widely considered for clinical applications, likely due to the focus on modulating 5-HT_2_ and 5-HT_3_ subtypes, and the scarcity of potent 5-HT_1A_ PAMs.

### Limitations

It is worth mentioning that despite the promising activity observed, the metabolism of (±)-CBL in both animal models and humans remains entirely unknown. As no metabolic enzymes were present in the in vitro media, we can conclude that the observed allosteric modulation was solely attributable to (±)-CBL itself rather than any potential metabolites. Cannabinoids are known for rapid metabolism, in some cases resulting in metabolites with distinct biological activities compared to the parent molecule.^55^ Consequently, identifying the metabolites of (±)-CBL and evaluating their biological effects, particularly at the 5-HT_1A_ receptor, represents an important avenue for future investigation. In addition, this study focuses on the β-arrestin pathway, given that serotonin acts as a nonbiased agonist of the 5-HT_1A_ receptor,^56^ engaging both β-arrestin and G-protein pathways equivalently. However, some PAMs of G-protein-coupled receptors GPCRs are reported to act as biased allosteric modulators (BAMs). For example, BMS-986122 functions as a biased PAM of morphine and DAMGO at the μ-opioid receptor (MOR), selectively enhancing G-protein activation.^57^ Such BAM properties have not yet been reported for the 5-HT_1A_ receptor. Future studies should thus consider examining the PAM activity of (±)-CBL in both cAMP and β-arrestin signaling to explore potential bias toward any specific pathway.

## Conclusion

In summary, the investigation of (±)-CBL presented in this study marks a significant advancement in our understanding of this unique phytocannabinoid. This study successfully improved the synthesis of (±)-CBL and reported its crystal structure and stability for the first time. Utilizing radioligand binding assays, we discovered that (±)-CBL does not exhibit significant affinity toward the cannabinoid receptors but shows notable interaction with the 5-HT_1A_ serotonin receptor, distinguishing it from CBD. The β-arrestin PathHunter assay further confirmed that (±)-CBL acts as a PAM of the 5-HT_1A_ receptor, enhancing serotonin-induced receptor activation significantly, particularly at high serotonin concentrations. The unique allosteric modulation profile of (±)-CBL at the 5-HT_1A_ receptor underscores its potential as a novel therapeutic agent. For instance, given the role of the 5-HT_1A_ receptor in neuromodulation and mental health, the potent PAM effect of (±)-CBL could pave the way for innovative drug discovery strategies that target this receptor subtype. Future research should focus on further elucidating the pharmacological properties of (±)-CBL, its safety profile, and its therapeutic potential *in vivo*.

## Experimental Section

### General Experimental Procedures

ZSM-5 (MR38), SSZ-13, and AlMCM-41 were purchased from ACS Materials (Pasadena, CA, US). The remaining chemicals for the synthesis stage were purchased from Sigma-Aldrich (St. Louis, MO, US). (±)-CBC was synthesized with olivetol and citral condensation, as reported previously.^58^ Analytical standards for HPLC analysis were provided by Cayman Chemicals (Ann Arbor, MI, US). Naturally occurring CBD was crystallized from CBD-dominant *Cannabis sativa L.* extract in heptane. [^3^H]-CP55940 and [^3^H]-8-Hydroxy-DPAT were provided by Dalriada Drug Discovery (Mississauga, ON, Canada). Eurofins Discovery (Fremont, CA, US) provided CP55940 (a mixture of two enantiomers) and serotonin for functional assays. (±)-CBL isolate (>98%) for stability studies was purchased from Sanobiotec (Vilniaus, Lithuania), and MCT oil was purchased from Kraft Chemical (Lake Zurich, IL US). (±)-CBL Certified Reference Material for *in vitro* studies was purchased from Cayman Chemicals (Ann Arbor, MI, US).

### Synthesis

A 50 mL test tube equipped with a magnetic stir bar was prepared. Into this tube, (±)-CBC (50 mg, 95% purity) and 50 mg of a heterogeneous catalyst were added. The tube was then sealed with a septum and purged with nitrogen gas (N_2_) to create an inert atmosphere. Subsequently, 10 mL of solvent was introduced into the tube via a syringe, ensuring the mixture remained under N_2_. The mixture was stirred under the conditions of time and temperature, as specified in Table 1. Postreaction, the catalyst was removed by filtration through filter paper and washed twice, each time with 10 mL of chloroform. The filtrate and wash solutions were combined, and the volume was reduced to approximately 10 mL using a rotary evaporator set at 50 °C. To induce (±)-CBL crystallization, the concentrated solution was cooled in a refrigerator. The crystallized (±)-CBL was collected by filtration using preweighed filter paper and washed twice with 10 mL of heptane. The filtered crystals were then dried in a room temperature vacuum chamber overnight. The yield of (±)-CBL was determined using an Agilent 1290 HPLC system (Santa Clara, CA, US), which was equipped with a validated method specifically for the quantification of cannabinoids.

### Crystallography

The crystal sample was mounted on a Mitegen polyimide micromount with a small amount of Paratone N oil. All X-ray measurements were made on a Bruker Kappa Axis Apex2 diffractometer at 110 K. The unit cell dimensions were determined from a symmetry-constrained fit of 9884 reflections with 6.0° < 2θ < 71.12°. The data collection strategy was a number of ω and φ scans, which collected data up to 71.346° (2θ). The frame integration was performed using SAINT (Bruker-AXS, Madison, WI, USA). The resulting raw data were scaled and absorption corrected using a multiscan averaging of symmetry equivalent data using SADABS (Bruker-AXS, Madison, WI, USA). The structures were solved using a dual space methodology using the SHELXT program.^59^ Most non-hydrogen atoms were obtained from the initial solution. The remaining atomic positions were obtained from subsequent difference Fourier maps. The hydrogen atoms were introduced at idealized positions and were allowed to ride on the parent atom. The n-penytl group exhibited a disorder where the chain adopted three different conformations. The normalized occupancies refined to values 0.488(2), 0.345(2), and 0.167(2). A graphic depiction of the disorder is given in Figure 1b. The structural model was fit to the data using full-matrix least-squares based on *F*^2^. The calculated structure factors included corrections for anomalous dispersion from the usual tabulation. The structure was refined using the SHELXL program from the SHELX suite of crystallographic software.^60^ Graphic plots were produced using the NRCVAX program suite.^61^

**Table 2:** Summary of Crystal Data for (±)-CBL Crystal Structure

Formula	C_21_H_30_O_2_
Formula weight (g/mol)	314.45
Crystal dimensions (mm)	0.433 × 0.222 × 0.114
Crystal color and habit	colorless prism
Crystal system	monoclinic
Space group	*P*2_1_/*n*
Temperature, K	110
*a*, Å	5.8756(14)
*b*, Å	23.937(5)
*c*, Å	12.972(3)
α, deg	90
β, deg	97.596(8)
γ, deg	90
*V*, Å^3^	1808.4(7)
Number of reflections to determine final unit cell	9884
Min and max 2θ for cell determination, deg	6.0, 71.12
*Z*	4
*F*(000)	688
ρ (g/cm)	1.155
λ, Å (Mo Kα)	0.71073
μ (cm^–1^)	0.072
Diffractometer type	Bruker Kappa Axis Apex2
Scan type(s)	phi and omega scans
Max 2θ for data collection, deg	71.346
Measured fraction of data	0.999
Number of reflections measured	80 885
Unique reflections measured	8373
*R*_merge_	0.0539
Number of reflections included in refinement	8373
Cut off threshold expression	*I* > 2σ(*I*)
Number of parameters in least-squares	278
*R*_1_^a^	0.0519
*wR*_2_	0.1314
*R*_1_ (all data)	0.0739
*wR*_2_ (all data)^b^	0.1449
GOF^c^	1.025
Maximum shift/error	0.001
Min and max peak heights on final DF map (*e*^*–*^/Å)	–0.266, 0.526

a *R*_1_ = *∑*(|*F*_o_| – |*F*_c_|)/*∑F*_o_.

b *wR*_2_ = [ *∑*(*w*(*F*_o_^2^ – *F*_c_^2^)^2^)/*∑*(*wF*_o_^4^) ]^1/2^.

c GOF = [ *∑*(*w*(*F*_o_^2^ – *F*_c_^2^)^2^)/(No. of reflns. – No. of params.) ]^1/2^.

### Stability Studies

Samples of (±)-CBL isolate were prepared for the stability study by weighing 200 mg of the isolate into ten individual amber 2 mL HPLC vials. The (±)-CBL solution samples were created by dissolving (±)-CBL isolate into MCT oil at room temperature to achieve a concentration of 50 mg/g. No heat was applied during this process; instead, the mixture was vigorously stirred for 3 days in a sealed amber vial under ambient conditions to ensure complete dissolution and suspension of the (±)-CBL in the MCT oil. Subsequently, 2 g of the mixture was dispensed into ten individual 10 mL amber vials. These vials were divided and stored in two separate temperature-controlled stability chambers, labeled as “real-time” (25 °C, 60% RH) and “accelerated” (40 °C, 75% RH). Samples were retrieved from the chambers at their designated intervals, every 2–4 weeks, and analyzed using HPLC-DAD with a validated method specifically designed for the quantification of cannabinoids. Each stability time point was measured from a single sample in three analytical replicates.

### Radioligand Binding Assay

First, the compound plate was prepared by creating eight different doses of reference compounds (CP55940 for CB_1_/CB_2_, and serotonin for 5-HT_1A_), starting from a 5 mM DMSO stock solution and performing 5-fold serial dilutions. Additionally, 10 mM and 1 mM DMSO stock solutions of (−)-CBD and (±)-CBL were prepared. A total of 750 nL of both the reference and test compounds was transferred to a 96-well compound plate, followed by the addition of 150 μL of assay buffer to each well to achieve a 5× final concentration. Each concentration measurement was conducted in triplicate. The plates were centrifuged at 1000 rpm for 30 s and then agitated at 600 rpm for 5 min at room temperature. Separately, 50 μL of 0.5% v/v PEI was added to each well of UniFilter-96 GF/C plates. These plates were sealed and incubated at 4 °C for 3CBD hours, then washed twice with ice-cold wash buffer. The cell membrane was diluted with assay buffer, and 330 μL was transferred to 96 round deep-well plates to reach a concentration of 10 μg per well. From the compound plate, 110 μL of two concentrations of CBD and (±)-CBL, as well as eight concentrations of reference compounds, were transferred to the 96 round deep-well plates. The radiolabeled ligand was diluted in assay buffer, and 110 μL of this solution was added to the 96 round deep-well plates to achieve a 5× final concentration of radioligand (10 nM [^3^H]-CP55940 for CB_1_/CB_2_ and 1 nM [^3^H]-8-Hydroxy-DPAT for 5-HT_1A_). The plates were centrifuged at 1000 rpm for 30 s and then agitated at 600 rpm for 5 min at room temperature. The plates were sealed and incubated at 30 °C for 90 min. The incubation was halted by vacuum filtration onto GF/C filter plates, followed by four washes with ice-cold wash buffer. The plates were then dried at 37 °C for 45 min. After adding 40 μL of scintillation cocktail, radioactivity signals were detected using a Microbeta2 microplate counter (PerkinElmer, Waltham, MA, US).

### PathHunter Assay

For the 5-HT_1A_ agonist assay, PathHunter β-arrestin cells (CHO-K1) were expanded from freezer stocks using standard procedures provided by Eurofins. The cell line manual was followed for cell growth, including details on cell culture media, supplementation, cell handling, and preparation. Briefly, cells were seeded at a total volume of 20 μL (10,000 cells) into white-walled, 384-well microplates and incubated at 37 °C for the appropriate time before testing. A stock acetonitrile solution was prepared at a 1 mg/mL concentration of (±)-CBL. Intermediate compound concentrations were created using a 10-point series of 3-fold serial dilutions in a compound dilution buffer on a separate dilution plate. Each dilution was prepared at 5× the final screening concentration. A total of 5 μL of the sample solution was added to the cells (resulting in the highest final concentration of 12 μM for the compound and 0.4% for acetonitrile) and incubated at 37 °C for 90 min in a 5% CO2 atm. Each concentration measurement was conducted in triplicate. Following this, 12.5 μL of Working Detection Solution was added, and the cells were incubated for 1 h at room temperature in the dark. The assay signal was generated by adding 15 μL (50% v/v) of the PathHunter Detection reagent cocktail, followed by another hour of incubation at room temperature. Finally, the microplates were read using a PerkinElmer Envision instrument (PerkinElmer, Waltham, MA, US) to detect the chemiluminescent signal.

For allosteric mode, cells were first preincubated with (±)-CBL, followed by a serotonin challenge at the EC_80_ or EC_20_ concentrations. Each (±)-CBL dilution was prepared at 10 times the intended final concentration. Then, 2.5 μL of these samples were added to the cells, resulting in a maximum final concentration of 12 μM for (±)-CBL and approximately 0.4% (v/v) acetonitrile. Each concentration measurement was conducted in triplicate. The assay plate was incubated at 37 °C with 5% CO_2_ for 30 min. Next, 2.5 μL of a serotonin stock solution (10 times the final concentration) was added to the cells, achieving a final concentration of 175 (EC_80_) or 40 nM (EC_20_). The EC_80_ and EC_20_ values for serotonin were previously determined by agonist assay. The cells were then incubated for 90 min at 37 °C with 5% CO_2_. To generate the assay signal, 12.5 μL of a working detection solution was added to the cells, which were then incubated for an hour at room temperature in the dark. Signal detection was performed using a PerkinElmer Envision (Waltham, MA, USA) instrument to measure chemiluminescence.

A more detailed assay protocol can be obtained from Eurofins (Catalog #: 93-0696E2CP0L).^62^

## Data Availability

CCDC 2379846 contains the supplementary crystallographic data for this paper, and these data can be obtained free of charge via www.ccdc.cam.ac.uk/data_request/cif, or by emailing data_request@ccdc.cam.ac.uk, or by contacting The Cambridge Crystallographic Data Centre, 12 Union Road, Cambridge CB21EZ, UK; fax: + 441223336033.

## Supporting Information Available

^1^H NMR and HPLC characterization data for (±)-CBL (PDF)
Illustrated crystallographic information for (±)-CBL (CIF)

## Supplementary Material

np4c00977_si_001.pdf

np4c00977_si_002.cif
