# Supplementary material for: An Unexpected Activity of a Minor Cannabinoid: Cannabicyclol (CBL) Is a Potent Positive Allosteric Modulator of Serotonin 5-HT1A Receptor
Source: J Nat Prod. 2025 Jan 15;88(1):58–66. doi: 10.1021/acs.jnatprod.4c00977 (PMC11774245; doi:10.1021/acs.jnatprod.4c00977)
Supplement: Supplementary file 1 — np4c00977_si_001.pdf [file np4c00977_si_001.pdf]

An unexpected activity of a minor cannabinoid: Cannabicyclol (CBL) is a potent positive allosteric modulator of serotonin 5-HT_{1A} receptor

Mehdi Haghdoost[1], Yvonne DePorre[2], Max Figi[2], Scott Young[2], Caitlyn Krebs[1] and Marcel O. Bonn-Miller[2]

[1] Nalu Bio Inc., 38 Keyes Avenue, Suite 117, San Francisco, CA 94129, USA

[2] Charlotte's Web, 700 Tech Court, Louisville, CO 80027, USA

** Correspondence: marcel.bonn-miller@charlottesweb.com*

Table of Contents

^{1}H NMR spectrum of (±)-CBL in $CDCl_3$ (400 MHz) S2

HPLC method S3

HPLC-DAD spectrum (215 nm) of (±)-CBL showing >97% purity S4

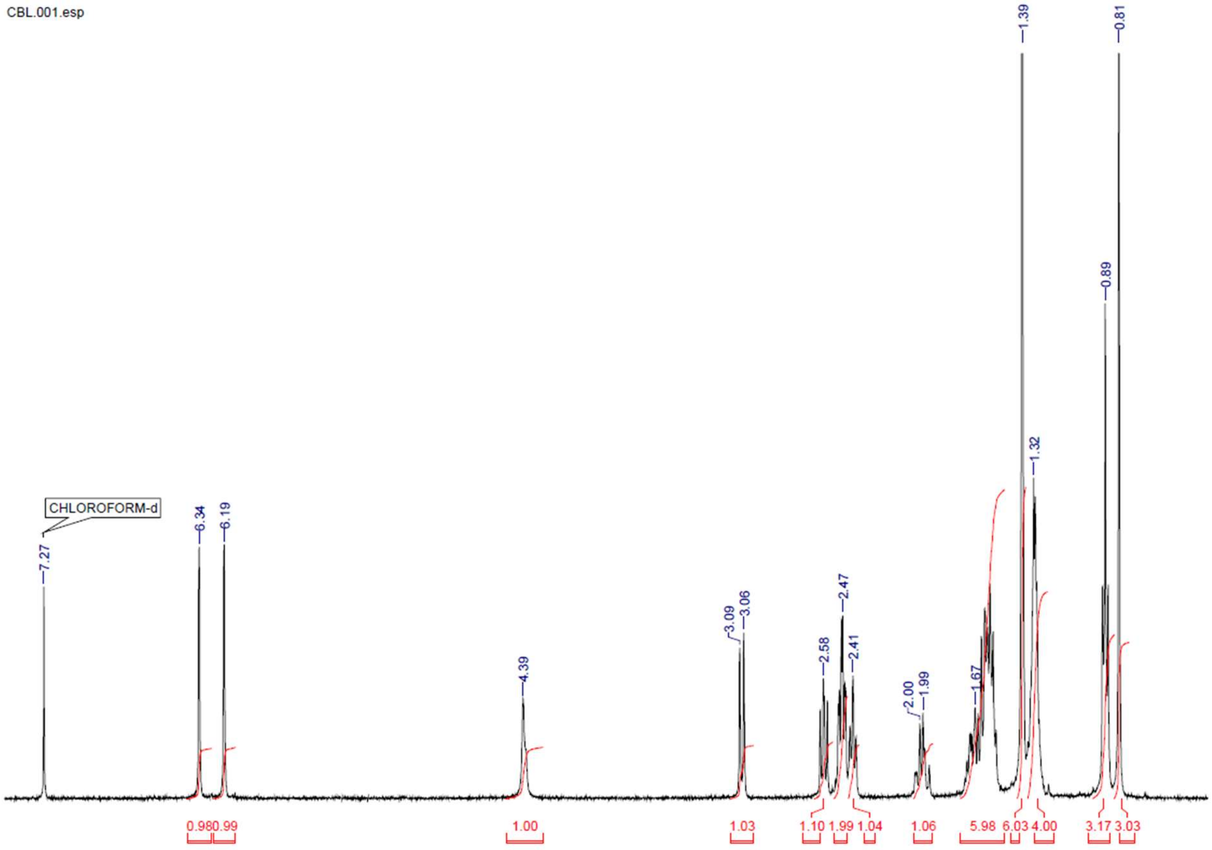

Fig S1. ^{1}H NMR spectrum of (±)-CBL in $CDCl_3$ (400 MHz).

HPLC method. A 1260/1290 Infinity II HPLC system (Santa Clara, California, US), equipped with a Poroshell 120 EC-C18 column (4.6 x 250 mm, 2.7 μm), was utilized to monitor the progress of synthesis and assess the purity and stability of the samples. Key parameters of the analytical method, such as mobile phases, flow rate, and gradients, are detailed in Table S1. The column temperature was maintained at 60 °C, and the DAD was set to detect at a wavelength of 215 nm. For sample preparation, 200 mg of the sample was accurately weighed using an analytical balance, and 10 mL of extraction solution (methanol:chloroform, 9:1) was added to achieve a 50-fold dilution. The mixture was vortexed for 1 minute to ensure complete dissolution, then further diluted with the extraction solution to achieve 250- and 4000-fold dilutions. After filtration through a 0.2-μm filter, 20 μL of each diluted sample was injected for analysis. For quantitative measurements, an 8-point calibration curve was constructed using certified cannabinoid reference materials obtained from Cayman Chemicals (Ann Arbor, Michigan, US).

Table S1. Details of the HPLC method parameters.

HPLC Mobile Phases					
**A**	MilliQ Water				
**B**	Methanol				
**C**	Acetonitrile + 0.1% (v/v) Phosphoric Acid				
**D**	MilliQ Water + 0.1% (v/v) Phosphoric Acid				
**Time (min)**	**A (%)**	**B (%)**	**C (%)**	**D (%)**	**Flow Rate (mL/min)**
0.00	0.0	0.0	63.0	37.0	1.500
2.00	0.0	0.0	68.8	31.2	1.500
4.70	0.0	0.0	76.8	23.2	1.300
6.70	0.0	0.0	82.8	17.2	1.500
9.50	0.0	0.0	89.5	10.5	1.850
11.00	0.0	0.0	100.0	0.0	---
12.00	0.0	0.0	100.0	0.0	2.150
12.50	0.0	0.0	63.0	37.0	2.150
14.00	0.0	0.0	63.0	37.0	1.500
14.50	0.0	0.0	63.0	37.0	1.500

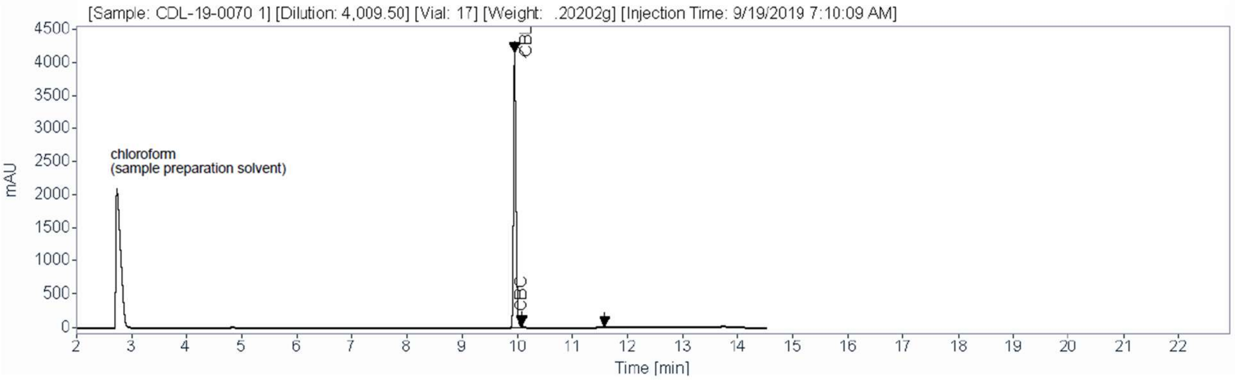

Fig S2. HPLC-DAD spectrum (215 nm) of (±)-CBL showing >97% purity.
